# Pulmonary nodular amyloidosis in a patient undergoing lobectomy: a case report

**DOI:** 10.1186/1752-1947-7-248

**Published:** 2013-11-07

**Authors:** Min Liu, Sen Wei, Xin Li, Hongyu Liu, Qinghua Zhou, Jun Chen

**Affiliations:** 1 Department of Lung Cancer Surgery, Tianjin Lung Cancer Institute, Tianjin Medical University General Hospital, Tianjin 30052, China

**Keywords:** Amyloidosis, Differential diagnosis, Pulmonary nodules

## Abstract

**Introduction:**

Pulmonary amyloidosis is rare and is often misdiagnosed due a lack of general awareness.

**Case presentation:**

In this case report we describe a 69-year-old Chinese woman who presented with a right lower lobe pulmonary nodule. After video-assisted thoracoscopic lobectomy, a histopathologic diagnosis of pulmonary nodular amyloidosis was rendered. She has done well postoperatively, showing no local recurrence or distal disease in an 8-month follow-up period.

**Conclusions:**

Distinguishing parenchymal nodular amyloidosis from a malignant lung tumor is often quite difficult. In the differential diagnosis of pulmonary nodules, nodular amyloidosis should be considered to avoid unnecessary lobectomy.

## Introduction

Amyloidosis is an uncommon disease whereby insoluble proteinaceous amyloid fibrils are deposited in bodily tissues. Only eight patients per million per year are affected. Localized pulmonary amyloidosis is an even less likely occurrence, incorporating vague clinical features and limited treatment options. Here we report the case a 69-year old woman with a pulmonary nodule of her right lower lobe that was suspicious of lung carcinoma. Ultimately, localized pulmonary amyloidosis was confirmed following video-assisted thoracoscopic (VAT) lobectomy.

## Case presentation

**Figure 1:**
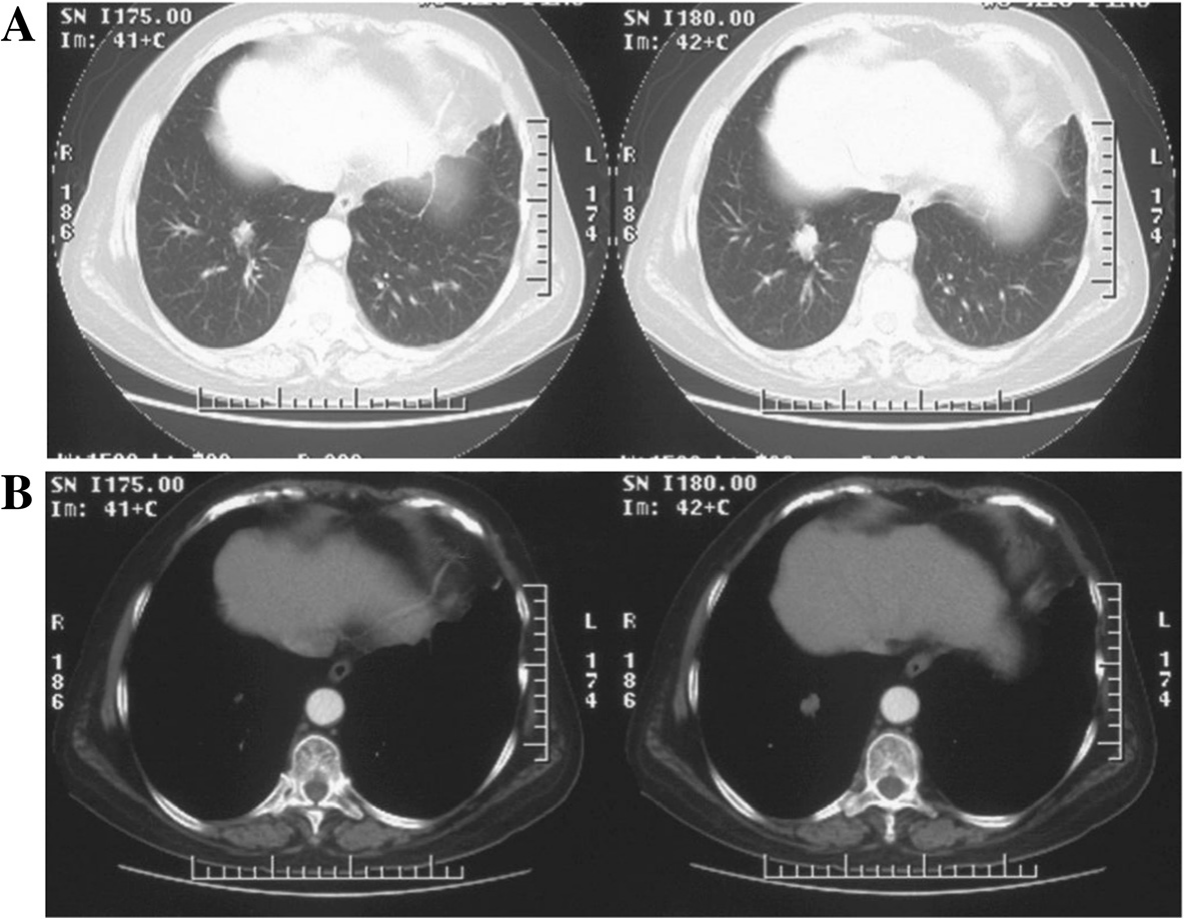
Computed tomography scans of chest: A, B) tumor-like mass with blurred contour and irregular margin shown in lung and mediastinal windows.

A 69-year-old Chinese woman was admitted for cough and expectoration (yellow phlegm) of about 2 weeks’ duration. These symptoms developed without apparent cause and in the absence of other problems, such as chest pain and/or distress, breathlessness, fever, hemoptysis, nausea, or vomiting. A computed tomography (CT) scan of her chest showed a rough-edged shadow nearly 24×18×30mm in the right lower lobe of her lung (Figure 1). She acknowledged several chronic disorders, including a 20-year history of bronchitis, recurrent urinary tract infections for 4 years, and abnormal pretibial skin pigmentation of 10-year duration involving both lower limbs. She denied smoking and had no family history of lung cancer. A review of her systems was noncontributory. The results of a peripheral blood count, baseline serum chemistry screening, and urinalysis were normal on admission, as were tumor biomarker tests and a purified protein derivative test for tuberculosis. A rough-edged shadow of her right lower lobe was again seen on an enhanced CT scan of her chest, although a CT scan of her abdomen, magnetic resonance imaging of her brain, and a bone scan were all normal. Her right bronchial tree also appeared normal on bronchoscopic examination, with no indication of malignancy in the biopsy and washings procured.

**Figure 2:**
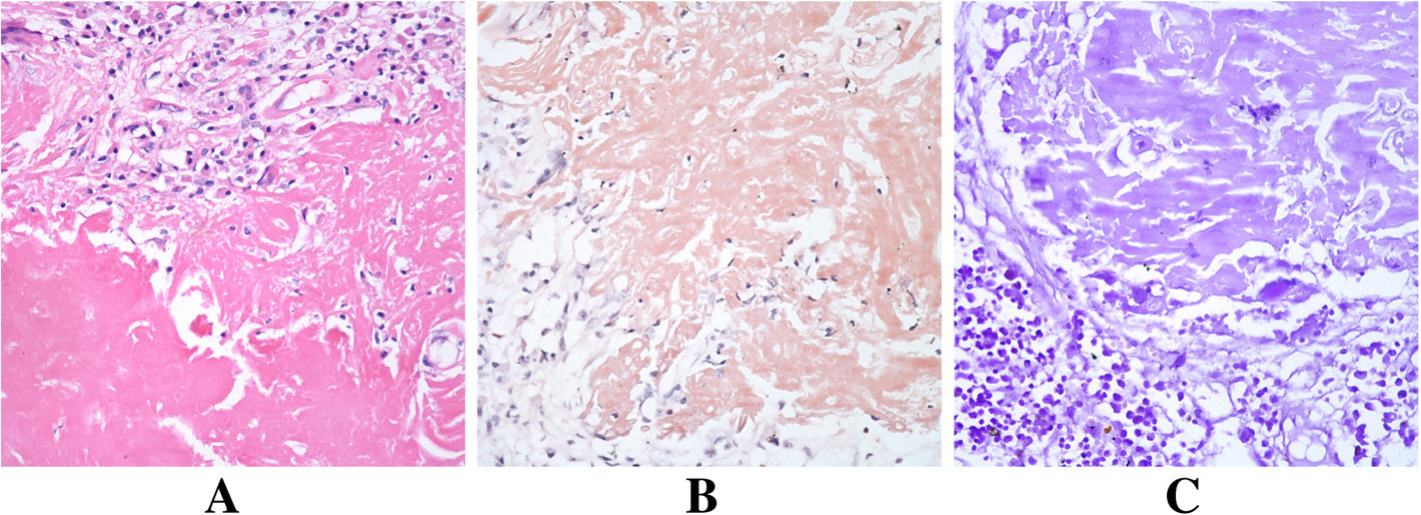
Histopathology (×200): A) routine hematoxylin and eosin stain of amyloid nodule; B, C) Congo red (pink) and methyl violet (purple) stains, positive for amyloid.

A right-sided video-assisted thoracoscopic (VAT) lower lobectomy and systematic mediastinal lymphadenectomy were performed. A tumor of her right lower lobe, roughly 3cm in diameter, was evident at surgery, with puckering of visceral pleura. Histopathologic examination later confirmed a diagnosis of nodular amyloidosis, supported by Congo red- and methyl violet-positive staining (Figure 2A-C). Bronchial and mediastinal lymph nodes were interpreted as reactive hyperplasia.

Her postoperative course was uneventful. She was discharged 4 days after surgery and showed no signs of local recurrence or distal disease at an 8-month follow-up visit.

## Discussion

Amyloidosis, first described by Virchow in 1854, encompasses a spectrum of clinical conditions with differing manifestations where abnormal extracellular deposition of amyloid is common [1]. Formerly grouped by etiology (primary versus secondary), it is now classified as either systemic or limited forms. Systemic disease typically is seen in patients suffering chronic suppuration, myeloma, or tuberculosis and may affect multiple organs (with exception of the brain) but is less apt to involve the lungs. However, the respiratory tract (that is, lungs and blood vessels) is commonly affected in localized disease. The published incidence of amyloidosis is eight patients per million per year [2]. A total of 89 patients were diagnosed with amyloidosis at Beijing Union Medical College Hospital between 1986 and 2005, 59 of whom had respiratory tract involvement [3].

Amyloidosis of the respiratory tract is subsequently divided into laryngeal, tracheobronchial, and parenchymal types, as noted by Gillmore and Hawkins [4]. Parenchymal disease is then further categorized as focal (nodular) or diffuse (interstitial). The patient reported here qualifies as parenchymal nodular amyloidosis (by imaging and histopathologic findings), also known as amyloid tumor for obvious reasons. Pulmonary amyloid nodules may occur singly or as multiple lesions that tend to occupy peripheral and subpleural areas of lower lobes [4]. This particular nodule was indeed found in the periphery of her right lower lobe. Such lesions are occasionally discovered by imaging or tissue biopsy and are usually asymptomatic.

According to the literature, parenchymal nodular amyloidosis is not linked with systemic disease but may persist for years, running a benign course (with reticulonodular pattern) [5]. On CT scan of the chest, four features are in favor of amyloid nodule: 1) sharp edges and lobulated contours; 2) calcifications (approximately 50%); 3) variability in shape and size (0.5 to 15cm); and 4) slow, non-regressive growth, rarely with cavitation [4,6]. However, none of these are specific for the disease.

Many of the above factors were in keeping with our patient. Although hospitalized for respiratory symptoms (cough, expectoration), chronic bronchitis, rather than the demonstrable amyloid nodule, may explain these complaints. It is difficult to diagnose these lesions, owing to their diverse clinical presentations and lack of definitive imaging properties. The imaging similarities with malignant lung tumors make differentiation especially difficult. A positron emission tomography (PET)-CT scan may help in distinguishing the two, given that amyloidosis for the most part is not fluorodeoxyglucose-avid. However, a negative result would not necessarily secure a diagnosis of amyloidosis, and PET-CT scans are costly. Routine fiberoptic bronchoscopy has a low diagnostic accuracy, but CT-guided percutaneous lung biopsy or transbronchial biopsy may afford better success in establishing a diagnosis [7]. Currently, the gold standard is histopathologic confirmation, marked by green birefringence of Congo red-stained tissue under cross-polarization [8].

In this instance, bronchoscopic washings and biopsy disclosed only chronic inflammation, and a CT-guided biopsy was refused by the patient due to the high risk of complications. The diagnosis of pulmonary nodular amyloidosis was ultimately achieved after VAT lobectomy, performed without intraoperative frozen section for two primary reasons: 1) puckering of visceral pleura was interpreted as a manifestation of malignancy, and 2) wide local excision would have proved challenging, given the location of the nodule (deep and close to hilar region).

Because pulmonary nodular amyloidosis is so uncommon, no randomized controlled trials are available for therapeutic insight. By consensus, however, asymptomatic patients may not require surgical intervention. Periodic follow-up with careful monitoring may instead suffice. For symptomatic patients, carbon dioxide laser ablation or surgery (bronchoscopic or open resection) may be used as warranted [9], although relapse is still possible [4]. In the past, low-dose external beam radiation has also conferred some benefit [10]. Regardless of the therapy chosen, we feel that close clinical vigilance is paramount in managing this disease.

## Conclusion

Distinguishing parenchymal nodular amyloidosis from malignant lung tumors is difficult due to imaging similarities. Although rare, pulmonary amyloidosis must be kept in mind, opting for intraoperative diagnosis if feasible to avoid unnecessary lobectomy.

## Consent

The patient granted written informed consent for publication of this manuscript and the accompanying images. A copy of the written consent is available for review by the Editor-in-Chief of this journal.

## Competing interests

The authors have no competing interests to declare.

## Authors’ contributions

ML collected all data and authored the manuscript. SW and XL were responsible for patient care and analysis of follow-up data. HL provided histopathologic confirmation. QZ participated in data analysis and manuscript revisions. JC performed the surgical procedure, also contributing to data analysis and shaping of the manuscript. All authors have read and approved the final manuscript.
